# Atherogenic Index of Plasma (AIP) as a Long‐Term Prognostic Factor Following CABG: Unveiling Insights From a Large‐Scale Tertiary Center Registry Analysis

**DOI:** 10.1002/hsr2.70616

**Published:** 2025-04-16

**Authors:** Abolfazl Salari, Parvin Kalhor, Ahmad Vakili‐Basir, Houshang Bavandpour Karvane, Mina Pashang, Mojgan Ghavami, Arash Jalali, Farshid Alaeddini, Farzad Masoudkabir

**Affiliations:** ^1^ Tehran Heart Center, Cardiovascular Diseases Research Institute Tehran University of Medical Sciences Tehran Iran; ^2^ Department of Epidemiology and Biostatistics, School of Public Health Tehran University of Medical Sciences Tehran Iran; ^3^ Cardiac Primary Prevention Research Center, Cardiovascular Diseases Research Institute Tehran University of Medical Sciences Tehran Iran

**Keywords:** atherogenic index of plasma, coronary artery disease, coronary artery bypass graft, major cardiovascular adverse event

## Abstract

**Background and Aims:**

The role of the atherogenic index of plasma (AIP) in predicting major adverse cerebro‐cardiovascular events (MACCE) after coronary artery bypass grafting (CABG) surgery has not been fully explored. The present study aims to investigate the prognostic value of AIP in predicting MACCE and its individual components following CABG.

**Methods:**

This is a large‐scale retrospective study conducted on patients who underwent isolated CABG. The primary outcomes were all‐cause mortality and MACCE, which included acute coronary syndrome (ACS), Cerebrovascular accident (CVA)/transient ischemia attack (TIA), revascularization, and all‐cause mortality. Proportional Hazard (PH) Cox regression, considering stabilized Inverse probability weightings (IPW), was conducted after verifying the PH assumption.

**Results:**

Totally, 23,432 patients analyzed with median 111.4‐month follow‐up duration. After weighting all variables, a higher AIP was associated with a significantly increased risk of MACCE (HR = 1.05; 95% CI: 1.01–1.09; *p* = 0.006). Furthermore, AIP was a significant predictor of the risk of revascularization (HR = 1.15; 95% CI: 1.01–1.30; *p* = 0.034) and ACS (HR = 1.09; 95% CI: 1.01–1.17; *p* = 0.020). However, AIP couldn't be a prognostic factor for all‐cause mortality and CVA.

**Conclusion:**

AIP predicts MACCE, revascularization, and ACS after CABG, serving as a readily accessible prognostic factor.

**Abbreviations**
ACS: acute coronary syndrome;
AIP: atherogenic index of plasma;
BMI: body mass index;
CABG: coronary artery bypass graft;
CAD: coronary artery disease;
CVA: cerebrovascular accident;
DLP: dyslipidemia;
EAS: European Atherosclerosis Society;
EF: ejection fraction;
ESC: European Society of Cardiology;
HDL‐C: high‐density lipoprotein cholesterol;
HTN: hypertension;
LDL‐C: low‐density lipoprotein cholesterol;
MACCE: major adverse cerebro‐cardiovascular events;
MACE: major adverse cardiac events
MI: myocardial infarction;
PCI: percutaneous coronary intervention;
RCS: restricted cubic spline;
sdLDL‐c: small dense LDL‐C;
SMD: standardized mean difference;
T2DM: type 2 diabetes mellitus;
TC: total cholesterol;
TG: triglycerides;
TIA: transient ischemic attack;

## 1 Introduction

Coronary artery disease (CAD) is a significant global health concern, accounting for a substantial proportion of mortality worldwide [1, 2]. Over seven decades, coronary artery bypass graft (CABG) surgery emerged as a standard treatment strategy, particularly for multi‐vessel disease, to revitalize myocardial perfusion [3]. Postoperative risk factor management is critical for mitigating major adverse cerebro‐cardiovascular events (MACCE). Dyslipidemia (DLP), characterized by elevated levels of low‐density lipoprotein cholesterol (LDL‐C), triglycerides (TG), total cholesterol (TC), or a reduced level of high‐density lipoprotein cholesterol (HDL‐C), is a well‐established risk factor for CAD development and detrimental postoperative outcomes [4, 5, 6]. While LDL‐C is the primary DLP factor in CAD progression and postoperative complications, the European Society of Cardiology (ESC) and European Atherosclerosis Society (EAS) guideline strongly promote the use of lipid‐lowering agents [7]. However, certain patients remain at elevated risk of recurrent cardiovascular events despite achieving optimal LDL‐C levels with lipid‐lowering drugs, suggesting that solely focusing on LDL‐C may be insufficient [8, 9]. To address this limitation, a novel marker, the atherogenic index of plasma (AIP), has been introduced. AIP, calculated as the logarithmically transformed ratio of TG concentration to HDL‐C (log [TG/HDL‐C]), has gained recognition for its ability to elucidate lipid‐associated cardiovascular risk. AIP is correlated with the size of lipoprotein particles, reflects plasma atherogenicity, and is also associated with the risk of CAD [10]. Numerous studies have demonstrated a link between AIP and outcomes of major health conditions related to atherosclerosis processes, including stroke, CAD, and peripheral artery disease, and as a risk factor for developing CAD [11, 12, 13, 14, 15, 16, 17, 18, 19].

Qin et al. pioneered assessing AIP's role in predicting outcomes for patients with CAD undergoing revascularization. The procedure was percutaneous coronary intervention (PCI), and the findings revealed that an increasing AIP was associated with a rise in MACCE [20].

CABG remains another crucial revascularization alternative. Despite the absence of prior literature on AIP's role in predicting outcomes of patients undergoing CABG surgery, the present study aims to investigate the impact of baseline AIP on long‐term MACCE and all‐cause mortality in post‐CABG patients. Preoperative TG and HDL‐C values can predict the outcomes of patients who undergo CABG [21, 22]. The potential benefit of AIP is that it combines TG and HDL‐C into a single metric, providing a more comprehensive assessment of patient outcomes.

## 2 Materials and Methods

### 2.1 Study Design and Patients' Population

This study employed a large‐scale, registry‐based retrospective design, encompassing patients who underwent isolated CABG surgery at our institution between March 2007 and March 2017. We extracted demographic data, prior medical history, surgical details, and outcomes from our center's adult cardiac surgery database [23, 24]. The inclusion criteria for the study were patients diagnosed with CAD who underwent isolated CABG. The exclusion criteria for the study were individuals who meet the following criteria: 1. Missing follow‐up data; 2. Incomplete preoperative lipid panel (TG or HDL‐C); 3. Severe renal impairment (glomerular filtration rate [GFR] < 15 ml/min/1.73 m^2^ or on dialysis); 4. History of prior cardiac surgery (CABG, valve surgery, or both); 5. Familial hypertriglyceridemia (TG > 1000 mg/dl).

The study adhered to the Declaration of Helsinki principles. The Ethics Committee of Tehran University of Medical Sciences approved this study and all patients had informed consent upon entering documentation in the research project (code of ethics: IR. TUMS.MEDICINE.REC.1402.697).

### 2.2 Baseline Assessment

We meticulously documented patients' demographic characteristics, including age and sex, as well as CAD risk factors, such as type 2 diabetes mellitus (T2DM), hypertension (HTN), DLP, family history of CAD, cigarette smoking, and opium consumption status. We also gathered information on prior medical conditions, including myocardial infarction (MI) and its interval to surgery, peripheral artery disease, valvular heart disease, chronic obstructive pulmonary disease, and cerebrovascular accident (CVA). Additionally, we collected essential variables such as preoperative LDL‐C, HDL‐C, TG, GFR, ejection fraction (EF), body mass index (BMI), and the status and type of surgery. TG and HDL‐C levels were obtained within 7 days before operation after 12 h of fasting. Subsequently, The AIP was calculated using the formula: Log [TG/HDL‐C].

Individuals with fasting blood glucose levels ≥ 126 mg/dL, HbA1C ≥ 6.5%, plasma glucose ≥ 200 mg/dL 2 h after consuming 75 grams of glucose, or random blood glucose ≥ 200 mg/dL were considered T2DM patients [25]. HTN was defined as systolic blood pressure ≥ 140 mmHg or diastolic blood pressure ≥ 90 mmHg [26]. DLP was diagnosed based on the following minimum levels: TC ≥ 240 mg/dL, LDL‐C ≥ 160 mg/dL, TG ≥ 200 mg/dL, HDL‐C ≤ 40 mg/dL in men, and HDL‐C ≤ 50 mg/dL in women [27]. Additionally, patients receiving medications for T2DM, HTN, or DLP, even if their levels were within normal ranges, were classified as positive for the respective condition. Patients were categorized into four groups based on their EF: severely reduced (≤ 30%), moderately reduced (30% < EF ≤ 40%), mildly reduced (40 < EF ≤ 50%), and preserved or normal EF ( > 50%). The AIP was calculated using the formula: Log [TG/HDL‐C].

### 2.3 Follow‐up and End‐Point Definition

Patients were closely monitored through regular clinic visits at 6 and 12 months after surgery and annually thereafter. For patients unable to attend in‐person appointments, trained physicians conducted phone interviews to assess their progress.

The primary outcomes of this study were all‐cause mortality and the occurrence of MACCE. MACCE was defined as the occurrence of any of the following events in the postoperative period: acute coronary syndrome (ACS), CVA/transient ischemic attack (TIA), revascularization, or all‐cause mortality. Each event that occurred first was considered a MACCE. Secondary outcomes included separate analyses of each component of MACCE.

### 2.4 Statistical Analysis

All statistical analyses were conducted and reported in this study, adhering to the “Guidelines for Reporting of Statistics for Clinical Research” and “Statistical Analyses and Methods in the Published Literature” (SAMPL) guidelines [28, 29]. In terms of descriptive statistics, either mean and standard deviation (SD) or median and interquartile range (IQR) boundaries were applied for continuous variables. For categorical variables, frequencies with their percentages were calculated. At two distinct AIP levels, an independent t‐test or Mann–Whitney test was utilized to compare continuous parameters. Additionally, either Pearson's Chi‐squared test or Fisher's exact test was used for categorical variables, depending on which test was considered acceptable. The restricted cubic spline (RCS) curve was utilized to define the cut‐off value of AIP. This method revealed a nonlinear association between continuous variables and outcomes [30]. This ability of the RCS curve allowed for a more accurate depiction of complex associations and reducing the risk of overfitting the data. These features of this method do not exist in linear models [31]. To determine the turning point of the AIP variable that changed its effect on MACCE, a RCS with three knots at the 25th, 50th, and 75th percentiles was employed. Based on the proposed cut‐off, the AIP variable was divided into two levels. The stabilized inverse probability weighting (IPW) technique was used to adjust for the effects of potential confounders suggested according to literature review on AIP levels. The variance inflation factor (VIF) was calculated for all covariates to evaluate multicollinearity in the multivariable analysis. Variables with a VIF less than 5 were considered to have minimal risk of multicollinearity [32]. The standardized mean difference (SMD) was utilized to assess the covariate balance graphically. Proportional hazard (PH) cox regression, considering IPWs, was carried out after checking the PH assumption. The results were presented as hazard ratio (HR) with confidence interval (CI). A P‐value of less than 0.05 was considered statistically significant.

A study can have minimal risk of bias due to missing data if it meets two conditions: 1. Missing values are less than 10%. 2. Data are missing randomly [33]. In this study, less than 8% of the data were missing; therefore, we used a complete case analysis. Nevertheless, to ensure robustness, we also performed sensitivity analyses using multiple imputation by chained equations (MICE) to confirm that our results were consistent.

Analyses were conducted using the R Statistical language (version 4.3.0; R Core Team, 2023), using the packages WeightIt (version 0.14.2; Greifer N, 2023), ggsurvfit (version 1.0.0; Sjoberg D et al., 2023) and survival (version 3.5.7; Therneau T, 2023) [34, 35, 36, 37].

## 3 Results

### 3.1 Baseline Characteristics

**Figure 1:**
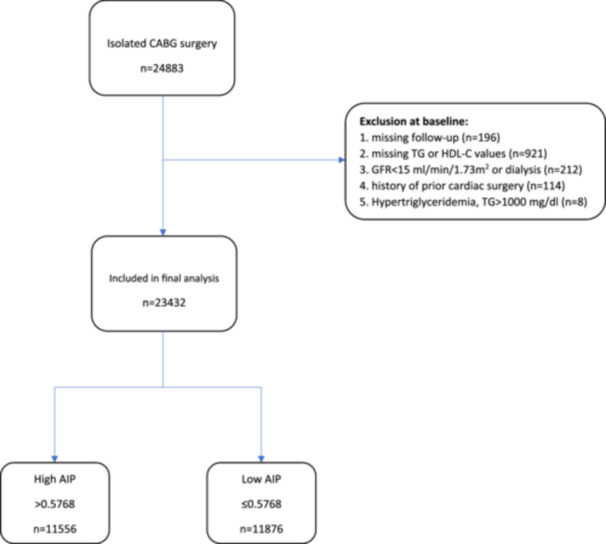
Flowchart of enrolled patients. AIP, atherogenic index of plasma; CABG, coronary artery bypass graft.

**Figure 2:**
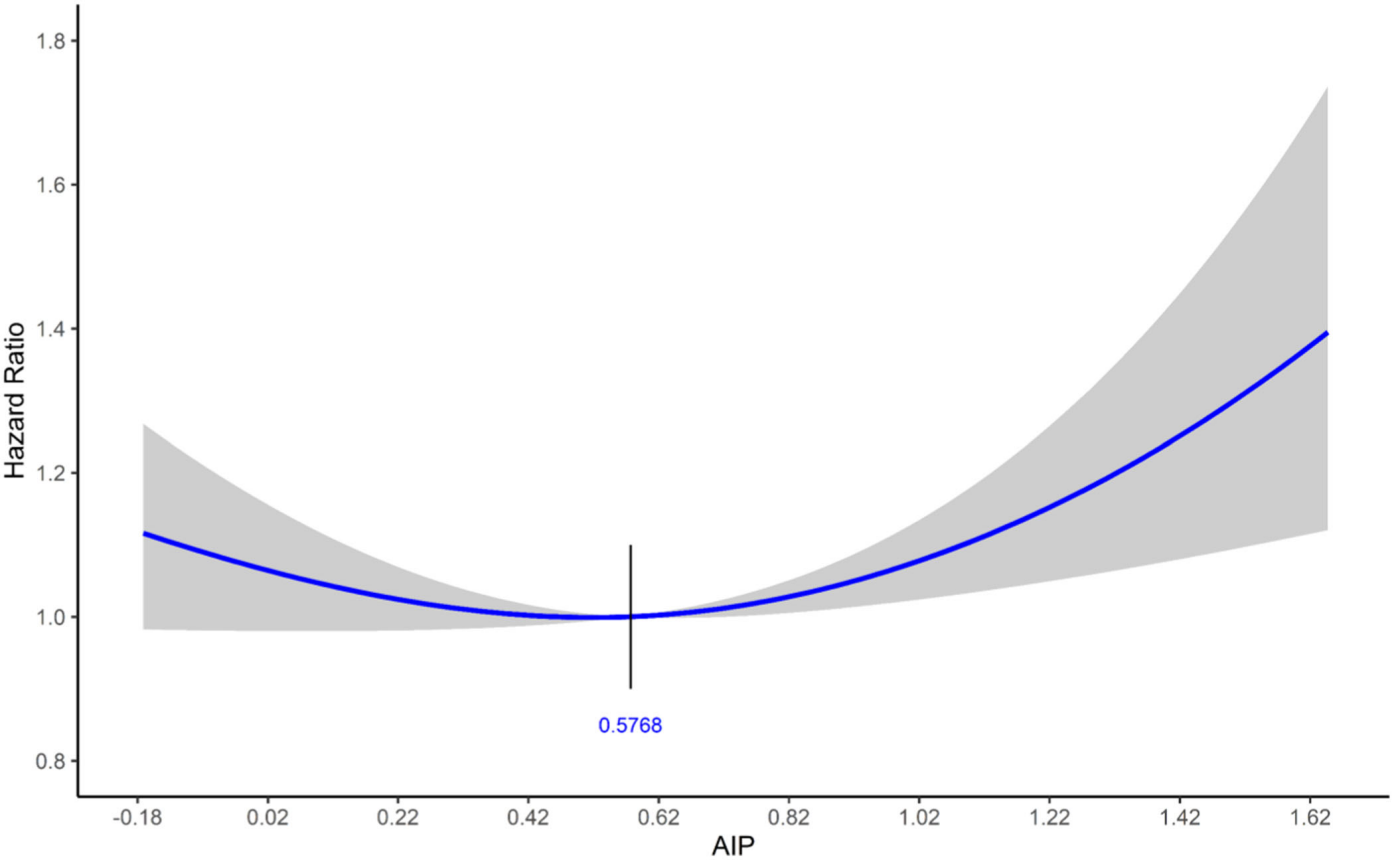
Restricted cubic spline plots on the atherogenic index of plasma (AIP) and major adverse cere‐bro‐cardiovascular event (MACCE).

A total of 23,432 patients were enrolled in this study. The study cohort's flow chart is illustrated in Figure 1. The median follow‐up duration was 111.4 months (95% CI: 110.1–112.3). The mean age of participants was 65.27 years, and 73.6% were male. A classification of patients into two groups based on AIP values was performed employing the RCS model (Figure 2). This model established 0.5768 as the appropriate cut‐off for AIP, with individuals possessing AIP values ≤ 0.5768 and > 0.5768 categorized as low and high AIP, respectively. Patients with high AIP (*n* = 11556, 49.3%) in comparison to those with low AIP (*n* = 11,876, 50.7%) were younger (63.96 vs 66.54, *p* < 0.001), had a higher prevalence of T2DM (44.1% vs 36.3%, *p* < 0.001), an increased occurrence of DLP (73.2% vs 56.9%, *p* < 0.001) and elevated level of LDL‐C (100.59 vs 92.00, *p* < 0.001). Detailed patient characteristics are presented in Table 1.

**Table 1:** Baseline characteristics according to AIP levels.

Characteristic	Overall, *N* = 23,432^1^	Low, *N* = 11,876^1^	High, *N* = 11,556^1^	*p*‐value
**Age**	65.27 (9.9)	66.54 (9.8)	63.96 (9.9)	**< 0.001**
**Gender**				0.876
Female	6185 (26.4%)	3140 (26.4%)	3045 (26.3%)	
Male	17,247 (73.6%)	8736 (73.6%)	8511 (73.7%)	
**Diabetes mellitus**	9399 (40.1%)	4306 (36.3%)	5093 (44.1%)	**< 0.001**
**Dyslipidemia**	15,224 (65.0%)	6760 (56.9%)	8464 (73.2%)	**< 0.001**
**Hypertension**	13,124 (56.0%)	6590 (55.5%)	6534 (56.6%)	0.106
**Family history**	9337 (39.9%)	4564 (38.5%)	4773 (41.3%)	**< 0.001**
**Opium**				0.319
No	19,795 (84.7%)	10,073 (85.0%)	9722 (84.3%)	
Current	2919 (12.5%)	1442 (12.2%)	1477 (12.8%)	
Former	662 (2.8%)	333 (2.8%)	329 (2.9%)	
**Cigarette smoking**				**< 0.001**
No	14,890 (63.7%)	7770 (65.5%)	7120 (61.7%)	
Current	4162 (17.8%)	1907 (16.1%)	2255 (19.6%)	
Former	4336 (18.5%)	2178 (18.4%)	2158 (18.7%)	
**Recent MI**				**0.004**
No	13,466 (58.2%)	6892 (58.8%)	6574 (57.6%)	
< 21 Days	4203 (18.2%)	2161 (18.4%)	2042 (17.9%)	
> = 21 Days	5463 (23.6%)	2660 (22.7%)	2803 (24.5%)	
**Prior angioplasty**	1560 (6.7%)	785 (6.6%)	775 (6.7%)	0.758
**PAD**	481 (2.1%)	262 (2.2%)	219 (1.9%)	0.091
**VHD**	4735 (20.5%)	2690 (23.0%)	2045 (18.0%)	**< 0.001**
**COPD**	865 (3.7%)	460 (3.9%)	405 (3.5%)	0.132
**Left main**	4650 (19.9%)	2487 (21.0%)	2163 (18.8%)	**< 0.001**
**CAG result**				**0.015**
SVD	980 (4.2%)	515 (4.3%)	465 (4.0%)	
2VD	5025 (21.5%)	2625 (22.1%)	2400 (20.8%)	
3VD	17,392 (74.3%)	8718 (73.5%)	8674 (75.2%)	
**Prior CVA/TIA**	1399 (6.0%)	692 (5.8%)	707 (6.1%)	0.346
**EF**				**0.018**
Normal	8794 (37.7%)	4498 (38.0%)	4296 (37.3%)	
Mildly reduced	8084 (34.6%)	4045 (34.2%)	4039 (35.1%)	
Moderately reduced	4631 (19.8%)	2305 (19.5%)	2326 (20.2%)	
Severely reduced	1844 (7.9%)	989 (8.4%)	855 (7.4%)	
**LDL‐C**	96.22 (36.7)	92.00 (35.3)	100.59 (37.5)	**< 0.001**
**BMI**				**< 0.001**
< 30	17,751 (76.0%)	9333 (78.8%)	8418 (73.0%)	
> = 30	5612 (24.0%)	2505 (21.2%)	3107 (27.0%)	
**GFR**				**< 0.001**
< 60	6062 (25.9%)	3409 (28.8%)	2653 (23.0%)	
> = 60	17,332 (74.1%)	8448 (71.2%)	8884 (77.0%)	
**Status of procedure**				0.060
Elective	22,152 (94.8%)	11,197 (94.5%)	10,955 (95.0%)	
Urgent and emergent	1225 (5.2%)	653 (5.5%)	572 (5.0%)	
**Off‐pump**	1764 (7.5%)	938 (7.9%)	826 (7.2%)	**0.030**

Abbreviations: BMI, body mass index; CAG, coronary angiography; COPD, chronic pulmonary obstructive disease; EF, ejection fraction; GFR, glomerular filtration rate; PAD, peripheral artery disease; SVD, single vessel disease; VHD, valvular heart disease; 2VD, 2 vessels disease; 3VD, 3 vessels disease.

^1^ *n* (%); Mean (SD).

### 3.2 Endpoints

In this study, 38.6% of patients experienced MACCE following CABG. This event occurred more frequently in the high AIP group compared to the low AIP group (39.0% vs 38.3%). When analyzing each MACCE component individually, they were more prevalent in the high AIP group, except for all‐cause mortality (Table 2). Supporting Information Table 1 provides patient characteristics based on their MACCE and all‐cause mortality experiences.

**Table 2:** Outcome frequencies in each group of AIP.

Characteristic	Overall, *N* = 23,432^1^	Low, *N* = 11,876^1^	High, *N* = 11,556^1^
MACCE	9055 (38.6%)	4552 (38.3%)	4503 (39.0%)
All‐cause mortality	5553 (23.7%)	2939 (24.7%)	2614 (22.6%)
ACS	3596 (15.3%)	1670 (14.1%)	1926 (16.7%)
CVA/TIA	1038 (4.4%)	510 (4.3%)	528 (4.6%)
Revascularization	1133 (4.8%)	491 (4.1%)	642 (5.6%)

^1^ n (%).

### 3.3 AIP and Outcomes

**Figure 3:**
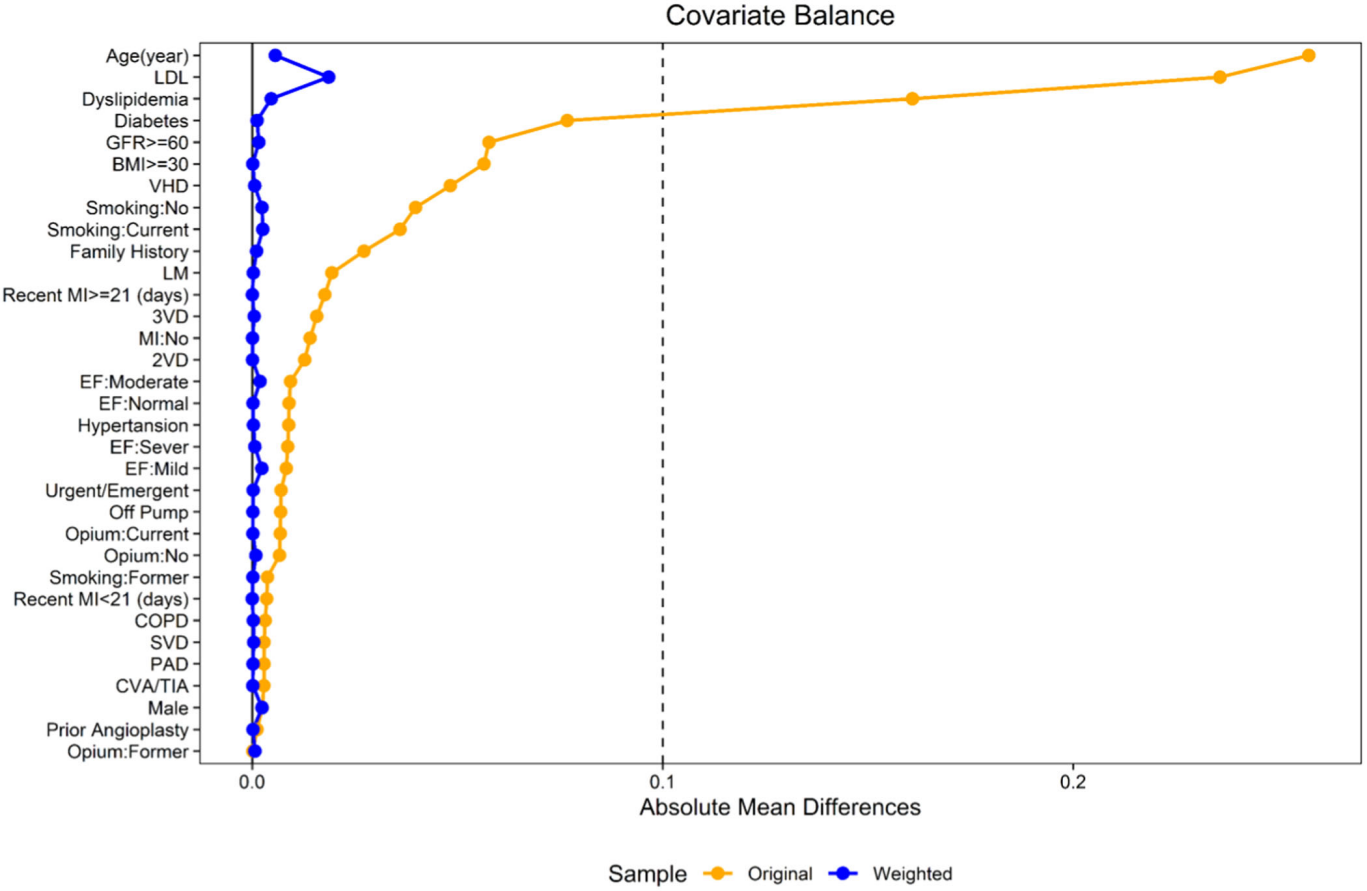
Covariate balance is measured by standardized mean difference before (original) and after weighting (Weighted). BMI, body mass index; CVA, cerebrovascular accident; COPD, chronic obstructive pulmonary disease; EF, ejection fraction; GFR, glomerular filtration rate; LM, left main; MI, myocardial infarction; PAD, peripheral arterial disease; SVD, single vessel disease; TIA, transient ischemic attack; VHD, valvular heart disease; 2VD, 2 vessels disease; 3VD, 3 vessels disease.

Figure 3 depicts the heterogeneity of the study population. The standardized difference compares the difference in means between AIP groups in units of SD. The most heterogeneous variables were age, LDL‐C, and DLP. Using the IPW method, we adjusted for these imbalances, resulting in an SMD < 0.1 for all variables. This indicates that the two groups of patients (high AIP and low AIP) became homogeneous after adjusting for confounding factors. All VIF values were below 3, indicating a low level of multicollinearity. Subsequently multicollinearity does not pose a significant concern for the analysis.

**Figure 4:**
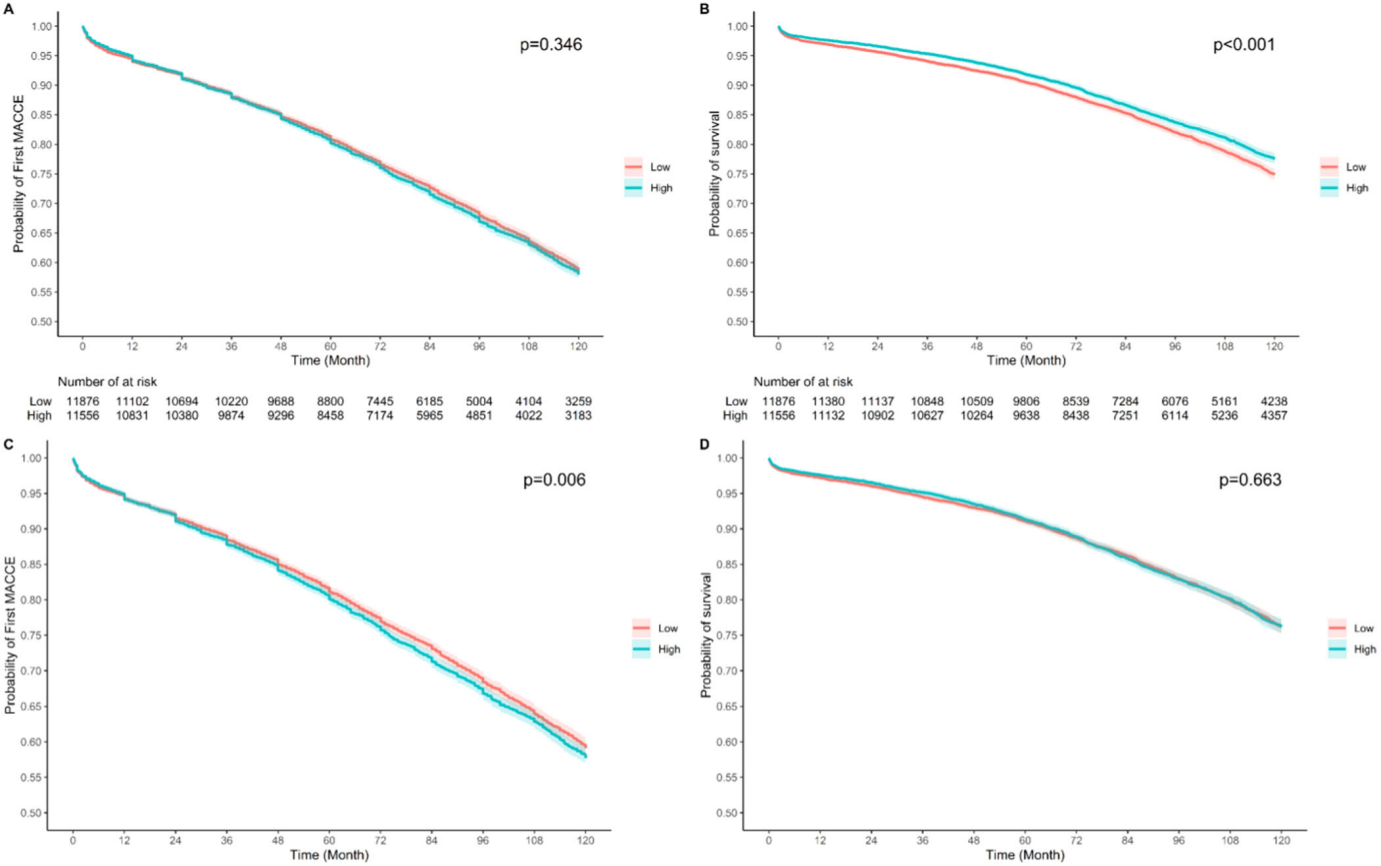
Kaplan‐Meier curves for comparison differences of outcomes between AIP groups. (A) MACCE before weighting (B) all‐cause mortality before weighting (C) MACCE after weighting (D) all‐cause mortality after weighting.

**Figure 5:**
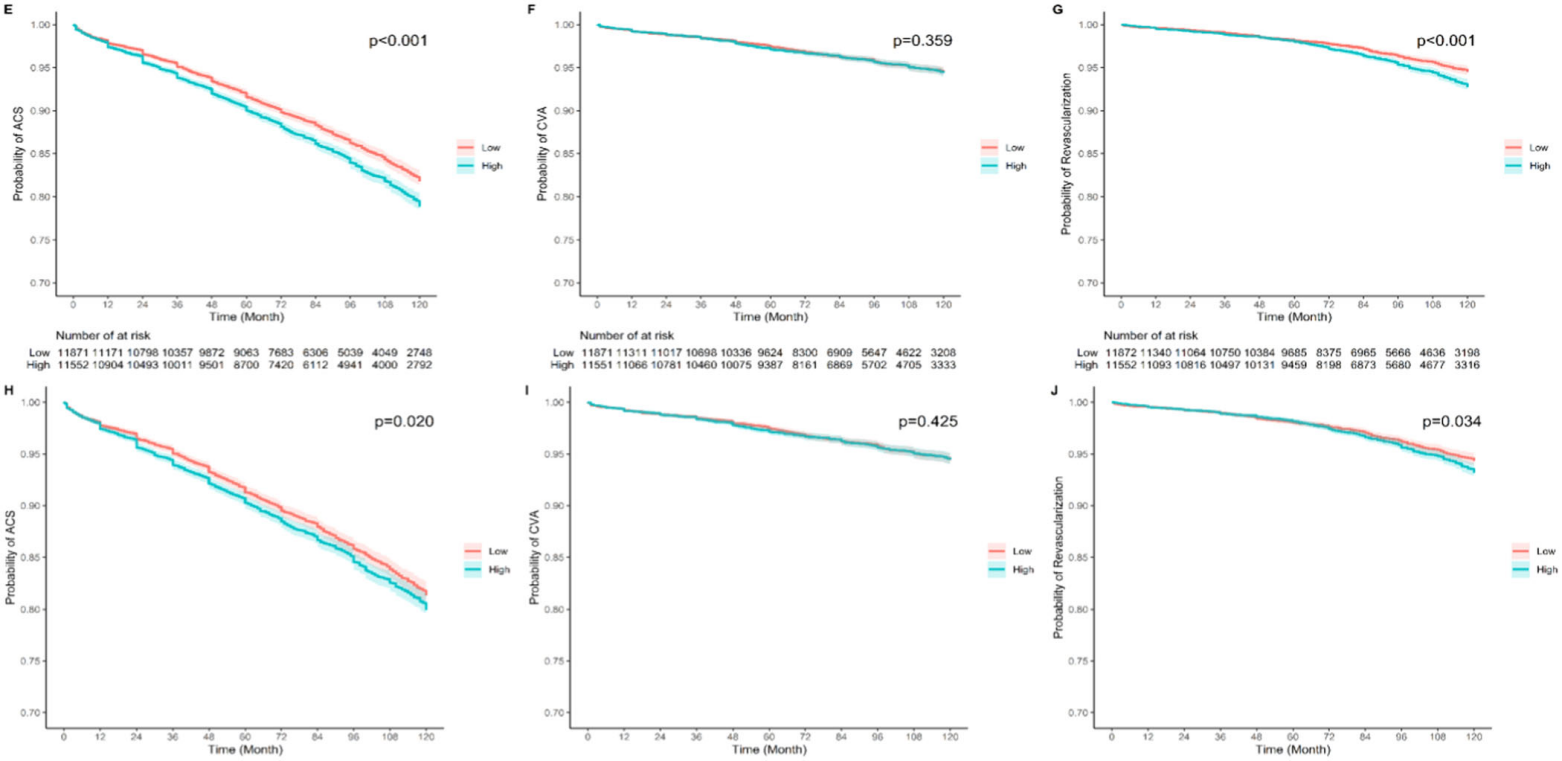
Kaplan‐Meier curves for comparison differences of outcomes between AIP groups. (E) ACS before weighting (F) CVA before weighting (G) revascularization before weighting (H) ACS after weighting (I) CVA after weighting (J) revascularization after weighting.

Table 3 and Figure 4 demonstrate that a rising AIP is associated with an increased risk of MACCE (HR: 1.05, 95% CI: 1.01–1.09, *p* = 0.006). However, AIP was not a significant prognostic factor for predicting all‐cause mortality (HR: 1.01, 95% CI: 0.96–1.07, *p* = 0.663). Additionally, increasing AIP puts patients at an increased risk of ACS and revascularization after CABG (Figure 5).

**Table 3:** The role of AIP to predict outcomes after CABG, before weighting, and after weighting by IPW methods.

	Before weighting			After weighting		
Characteristic	HR	95% CI	*p*‐value	HR	95% CI	*p*‐value
**MACCE**						
Low	—	—				
High	1.02	0.98, 1.06	0.342	1.05	1.01, 1.09	**0.006**
**ACS**						
Low	—	—		—	—	
High	1.19	1.11, 1.27	**< 0.001**	1.09	1.01, 1.17	**0.020**
**CVA/TIA**						
Low	—	—		—	—	
High	1.06	0.94, 1.20	0.360	1.05	0.93, 1.20	0.425
**Revascularization**						
Low	—	—		—	—	
High	1.33	1.18, 1.49	**< 0.001**	1.15	1.01, 1.30	**0.034**
**All‐cause mortality**						
Low	—	—		—	—	
High	0.89	0.84, 0.94	**< 0.001**	1.01	0.96, 1.07	0.663

Abbreviations: CI, confidence interval; HR, hazard ratio.

These findings suggest that AIP is a significant predictor of MACCE in patients undergoing CABG, while its association with all‐cause mortality requires further investigation.

## 4 Discussion

In this study, we investigated the prognostic value of AIP in patients who underwent CABG. The primary finding of this study demonstrated that high AIP independently predicted an increased risk of MACCE after CABG surgery. This association was evident even after controlling for other relevant factors. A more detailed analysis revealed that the elevated MACCE risk was primarily attributed to the increased incidence of ACS and revascularization procedures after CABG in patients with high AIP. These findings suggest that preoperative AIP levels could serve as a valuable biomarker for identifying patients at higher risk of ACS and revascularization after CABG, potentially guiding personalized management strategies. However, our study findings indicate that AIP has a limited ability to predict CVA and all‐cause mortality in this patient population. The mechanism behind all‐cause mortality and CVA involves a broader spectrum of underlying conditions, including inflammation, other comorbidities, and genetic susceptibilities, whereas AIP only reflects lipid metabolism status. In other studies, AIP has failed to predict all‐cause mortality and CVA, which is consistent with our findings [10, 38]. Another study conducted on 14,063 participants from the National Health and Nutrition Examination Survey (NHANES) database in the general population demonstrated that there is no association between high AIP and an increased risk of all‐cause mortality and cardiovascular mortality [39]. Although AIP's predictive power for CVA and all‐cause mortality is weak, it could still be a prognostic factor for predicting MACCE, ACS, and revascularization.

Recent studies have highlighted that increasing TG level or decreasing HDL‐C worsens CABG patients' outcomes [21, 40, 41]. While LDL‐C, another key component of DLP, is a recognized important risk factor for atherosclerosis, it is often within the normal range in patients with T2DM despite their abnormal lipid metabolism [20, 42]. Therefore, in patients with normal LDL‐C levels, recurrent cardiovascular events remain a significant concern [8, 9]. These observations emphasize the need for a more comprehensive index to assess dyslipidemia and its association with atherosclerotic risk. This led to the development of the AIP calculated as the logarithmic transformation of TG/HDL‐C (log [TG/HDL‐C]).

Small dense LDL‐C (sdLDL‐C), a subgroup of LDL‐C, is particularly atherogenic due to its enhanced susceptibility to oxidation and reduced affinity for LDL receptor‐mediated clearance, leading to increased cholesterol accumulation in the bloodstream [20, 43]. Recent studies indicate that sdLDL‐C is the most atherogenic lipoprotein factor, and an increase in this value is associated with a higher incidence of MACCE after PCI [44, 45]. Nonetheless, the lack of a cost‐effective and widely available method for assessing sdLDL‐C limits its clinical applicability [46]. Tomo et al. demonstrated a positive correlation between AIP and sdLDL‐C levels, suggesting that AIP might serve as a surrogate marker for sdLDL‐C [47]. Dobiasova et al. further strengthened this association by showing a negative correlation between AIP and LDL‐C particle size [48]. These findings imply that an elevated AIP value may reflect a higher proportion of atherogenic sdLDL‐C particles, exacerbating the atherosclerotic process. The association with sdLDL‐C and incorporation of TG and HDL‐C provide the rationales for utilizing AIP for predicting atherosclerotic‐related adverse events following CABG.

A study conducted by Qin et al. [20] in 2020 involved 2356 T2DM patients who underwent PCI and were followed up for 4 years. AIP was categorized using cut‐off points of 0.318. Cardiac death, MI, stroke, and revascularization were considered as MACCE. The MACCE in low and high AIP groups were 16.2% and 27%, respectively. The all‐cause mortality rate was also higher in the high AIP group (4.3% vs 2.3%). After adjusting AIP for age, BMI, sex, medical history, and treatment, it was found that AIP is an independent prognostic factor for predicting MACCE and all‐cause mortality after PCI. The HR values for MACCE and all‐cause mortality were 1.64 (95% CI: 1.36–1.97, *p* < 0.001) and 1.69 (95% CI: 1.05–2.72; *p* = 0.03), respectively. Additionally, AIP could be a prognostic factor for each component of MACCE except for stroke. It is important to note that the rates of mortality in our study may vary significantly compared to other studies. This is because this study was conducted on CABG patients while Qin et al.'s study evaluated PCI patients, and the patients were followed up for a longer time in our study, which could explain the higher mortality rate.

Wang and colleagues carried out a study on 1133 patients who had LDL‐C levels lower than 1.8 mmol/L and underwent PCI [10]. The patients were monitored for a median follow‐up period of 26 months, and the researchers used the same definition of MACCE as in Qin et al.'s study. After adjusting for other variables, the study found that patients with high AIP levels were more likely to experience MACCE and require revascularization. The HR for MACCE was 1.62 (95% CI:1.04–2.53; *p* = 0.03), while the HR for revascularization was 1.74 (95% CI: 1.06–2.87; *p* = 0.03). However, AIP was not found to be a good predictor of the risk of MI, stroke, all‐cause mortality, and cardiac death. This study highlights the importance of AIP levels in predicting outcomes after PCI in patients with tight control of LDL‐C.

In a study conducted on 798 T2DM patients who underwent PCI and were followed up for a median of 927 days, MACCE (a composite of all‐cause mortality, stroke, MI, and revascularization) was analyzed [49]. The patients were divided into four groups based on AIP quartiles. During the follow‐up, 24.8% of the patients had MACCE. The risk of MACCE doubled from Q1 to Q4 groups (Q4: 66 [33.2%]; Q1: 33 [16.2%]). Multivariate analysis showed that AIP is an independent factor for predicting adverse events after PCI. Rising AIP from Q1 to Q4 group is associated with more than twofold risk of MACCE (HR: 2.25; 95% CI: 1.44–3.52; *p* < 0.001). However, there was no significant increase in risk between the Q2 and Q1 groups or the Q3 and Q1 groups. It is recommended to divide patients into two groups with more accurate methods such as RCS. In this study, analysis that adjusted other variables wasn't done for each component of MACCE. Kaplan‐Meier curves for MACCE, which included all‐cause mortality, MI, CVA, and revascularization, demonstrated that only the Q4 group had a significant increase in revascularization, which means that the main reason for rising MACCE in the Q4 group depends on revascularization.

Zheng et al. [38] designed a study to investigate the predictive role of AIP in major adverse cardiac events (MACE) among nondiabetic patients who underwent PCI. The study considered cardiac death, revascularization, and MI as the components of MACE. The study followed up on a total of 5538 nondiabetic patients for an average of 28 months. Patients were divided into three groups based on AIP tertile. Multivariate analysis demonstrated that a rise in AIP was significantly associated with increasing MACE (third tertile vs. first: HR: 1.36; 95% CI: 1.01–1.82; *p* = 0.04). Further analysis of each component revealed that AIP could independently predict cardiac death due to MI, revascularization, and MI itself. Nevertheless, it doesn't possess this ability for all‐cause mortality and stroke.

To the best of our understanding, our study represents the first in‐depth investigation of AIP's predictive power for MACCE in patients undergoing CABG. Our findings corroborate previous studies conducted on PCI patients, consistently demonstrating an association between rising AIP levels and an increasing risk of MACCE and revascularization. This association aligns with the heightened susceptibility of atherosclerotic plaques to rupture in the presence of high AIP [10]. Notably, our study aligns with the broader body of evidence suggesting AIP's limited ability to predict all‐cause mortality. While some studies have indicated a weak or no association between AIP and all‐cause mortality, our results reinforce this notion. The association between AIP and MI remains a subject of debate, but our findings suggest a higher incidence of ACS in high AIP groups. Additionally, the impact of AIP on stroke outcomes following CABG or PCI remains inconclusive across various studies.

### 4.1 Study Strengths and Limitations

This study represents a pioneering effort to examine the predictive role of AIP in MACCE following CABG. Our study boasts several strengths, including a large patient cohort and extensive follow‐up duration. However, it also presents certain limitations. Notably, the database lacked information regarding lipid‐lowering agents, particularly statins, which could influence AIP levels and MACCE outcomes. Moreover, the absence of death certificate data precluded the determination of cardiac death, limiting the assessment of cardiovascular mortality specifically. As a result, we could only assess all‐cause mortality. Furthermore, the potential confounding effect of thyroid function on AIP levels was not considered in our analysis. Additionally, AIP could not be a predictor of all‐cause mortality and CVA following CABG. Also, this study included Iranian patients and the results may not generalize to other ancestries. This limitation could potentially influence the interpretation of our findings. Future studies should incorporate thyroid function assessment to address this issue and provide a more comprehensive understanding of AIP's impact on MACCE after CABG.

## 5 Conclusion

Elevating AIP levels emerge as a powerful predictor of MACCE, ACS, and revascularization after CABG, highlighting its role as a key indicator of atherosclerotic progression. Incorporating AIP into patient management could enhance risk stratification and optimize post‐CABG outcomes.

## Author Contributions

All authors have read and approved the final version of the manuscript. Farzad Masoudkabir had full access to all of the data in this study and takes complete responsibility for the integrity of the data and the accuracy of the data analysis.

## Ethics Statement

The Ethics Committee of Tehran University of Medical Sciences approved this study and all patients had informed consent upon entering documentation in the research project (code of ethics: IR. TUMS. MEDICINE. REC.1402.697).

## Consent

The authors have nothing to report.

## Conflicts of Interest

The authors declare no conflicts of interest.

## Transparency Statement

The lead author Farzad Masoudkabir affirms that this manuscript is an honest, accurate, and transparent account of the study being reported; that no important aspects of the study have been omitted; and that any discrepancies from the study as planned (and, if relevant, registered) have been explained.

## Supporting information


**Supplementary 1.** Baseline Characteristics according to MACCE and all‐cause mortality.

## Data Availability

The authors have nothing to report.
