# Supplementary material for: Atherogenic Index of Plasma (AIP) as a Long‐Term Prognostic Factor Following CABG: Unveiling Insights From a Large‐Scale Tertiary Center Registry Analysis
Source: Health Sci Rep. 2025 Apr 16;8(4):e70616. doi: 10.1002/hsr2.70616 (PMC12003921; doi:10.1002/hsr2.70616)
Supplement: Supplementary file 1 — Supplementary 1. Baseline Characteristics according to MACCE and all‐cause mortality. [file HSR2-8-e70616-s001.docx]

**Supplementary 1.** Baseline Characteristics according to MACCE and all-cause mortality

|  | All-cause mortality | | | MACCE | |
| --- | --- | --- | --- | --- | --- |
| **Characteristic** | **Overall**, N = 23,432^1^ | **Survived**, N = 17,879^1^ | **Deceased**, N = 5,553^1^ | **No**, N = 14,377^1^ | **Yes**, N = 9,055^1^ |
| **Age** | 65.27 (9.9) | 64.01 (9.7) | 69.30 (9.6) | 64.34 (9.6) | 66.75 (10.3) |
| **Gender** |  |  |  |  |  |
| *Female* | 6,185 (26.4%) | 4,618 (25.8%) | 1,567 (28.2%) | 3,524 (24.5%) | 2,661 (29.4%) |
| *Male* | 17,247 (73.6%) | 13,261 (74.2%) | 3,986 (71.8%) | 10,853 (75.5%) | 6,394 (70.6%) |
| **Diabetes Mellitus** | 9,399 (40.1%) | 6,698 (37.5%) | 2,701 (48.6%) | 5,274 (36.7%) | 4,125 (45.6%) |
| **Dyslipidemia** | 15,224 (65.0%) | 11,646 (65.1%) | 3,578 (64.4%) | 9,173 (63.8%) | 6,051 (66.8%) |
| **Hypertension** | 13,124 (56.0%) | 9,702 (54.3%) | 3,422 (61.7%) | 7,703 (53.6%) | 5,421 (59.9%) |
| **Family history** | 9,337 (39.9%) | 7,401 (41.4%) | 1,936 (34.9%) | 5,792 (40.3%) | 3,545 (39.2%) |
| **Opium** |  |  |  |  |  |
| *No* | 19,795 (84.7%) | 15,157 (85.0%) | 4,638 (83.7%) | 12,156 (84.8%) | 7,639 (84.5%) |
| *Current* | 2,919 (12.5%) | 2,147 (12.0%) | 772 (13.9%) | 1,734 (12.1%) | 1,185 (13.1%) |
| *Former* | 662 (2.8%) | 533 (3.0%) | 129 (2.3%) | 450 (3.1%) | 212 (2.3%) |
| **Cigarette smoking** |  |  |  |  |  |
| *No* | 14,890 (63.7%) | 11,374 (63.7%) | 3,516 (63.4%) | 9,131 (63.6%) | 5,759 (63.7%) |
| *Current* | 4,162 (17.8%) | 3,250 (18.2%) | 912 (16.5%) | 2,624 (18.3%) | 1,538 (17.0%) |
| *Former* | 4,336 (18.5%) | 3,221 (18.0%) | 1,115 (20.1%) | 2,592 (18.1%) | 1,744 (19.3%) |
| **Recent MI** |  |  |  |  |  |
| *No* | 13,466 (58.2%) | 10,566 (59.8%) | 2,900 (52.9%) | 8,609 (60.7%) | 4,857 (54.3%) |
| *<21 Days* | 4,203 (18.2%) | 3,167 (17.9%) | 1,036 (18.9%) | 2,574 (18.1%) | 1,629 (18.2%) |
| *>=21 Days* | 5,463 (23.6%) | 3,922 (22.2%) | 1,541 (28.1%) | 3,005 (21.2%) | 2,458 (27.5%) |
| **Prior angioplasty** | 1,560 (6.7%) | 1,213 (6.8%) | 347 (6.3%) | 934 (6.5%) | 626 (6.9%) |
| **PAD** | 481 (2.1%) | 267 (1.5%) | 214 (3.9%) | 209 (1.5%) | 272 (3.0%) |
| **VHD** | 4,735 (20.5%) | 3,185 (18.1%) | 1,550 (28.3%) | 2,692 (19.0%) | 2,043 (23.0%) |
| **COPD** | 865 (3.7%) | 563 (3.2%) | 302 (5.5%) | 450 (3.1%) | 415 (4.6%) |
| **Left main** | 4,650 (19.9%) | 3,441 (19.3%) | 1,209 (21.9%) | 2,852 (19.9%) | 1,798 (19.9%) |
| **CAG result** |  |  |  |  |  |
| *SVD* | 980 (4.2%) | 821 (4.6%) | 159 (2.9%) | 644 (4.5%) | 336 (3.7%) |
| *2VD* | 5,025 (21.5%) | 4,053 (22.7%) | 972 (17.5%) | 3,240 (22.6%) | 1,785 (19.7%) |
| *3VD* | 17,392 (74.3%) | 12,982 (72.7%) | 4,410 (79.6%) | 10,475 (73.0%) | 6,917 (76.5%) |
| **Prior CVA/TIA** | 1,399 (6.0%) | 927 (5.2%) | 472 (8.5%) | 745 (5.2%) | 654 (7.2%) |
| **EF** |  |  |  |  |  |
| *Normal* | 8,794 (37.7%) | 7,188 (40.3%) | 1,606 (29.1%) | 5,728 (40.0%) | 3,066 (34.0%) |
| *Mildly reduced* | 8,084 (34.6%) | 6,310 (35.4%) | 1,774 (32.1%) | 5,073 (35.4%) | 3,011 (33.4%) |
| *Moderately reduced* | 4,631 (19.8%) | 3,266 (18.3%) | 1,365 (24.7%) | 2,658 (18.5%) | 1,973 (21.9%) |
| *Severely reduced* | 1,844 (7.9%) | 1,061 (6.0%) | 783 (14.2%) | 874 (6.1%) | 970 (10.8%) |
| **LDL-C** | 96.22 (36.7) | 96.34 (36.6) | 95.81 (36.9) | 95.82 (36.6) | 96.85 (36.9) |
| **BMI** |  |  |  |  |  |
| *<30* | 17,751 (76.0%) | 13,496 (75.7%) | 4,255 (76.9%) | 10,945 (76.3%) | 6,806 (75.4%) |
| *>=30* | 5,612 (24.0%) | 4,335 (24.3%) | 1,277 (23.1%) | 3,393 (23.7%) | 2,219 (24.6%) |
| **GFR** |  |  |  |  |  |
| *<60* | 6,062 (25.9%) | 3,662 (20.5%) | 2,400 (43.3%) | 2,898 (20.2%) | 3,164 (35.0%) |
| *>=60* | 17,332 (74.1%) | 14,189 (79.5%) | 3,143 (56.7%) | 11,457 (79.8%) | 5,875 (65.0%) |
| **Status procedure** |  |  |  |  |  |
| *Elective* | 22,152 (94.8%) | 17,045 (95.5%) | 5,107 (92.2%) | 13,783 (96.1%) | 8,369 (92.7%) |
| *Urgent & emergent* | 1,225 (5.2%) | 795 (4.5%) | 430 (7.8%) | 564 (3.9%) | 661 (7.3%) |
| **Off-pump** | 1,764 (7.5%) | 1,409 (7.9%) | 355 (6.4%) | 1,214 (8.5%) | 550 (6.1%) |
| ^1^n (%); Mean (SD) | | | | | |

PVD: peripheral artery disease; VHD: valvular heart disease; COPD: chronic pulmonary obstructive disease; CAG: coronary angiography; SVD: single vessel disease; 2VD: 2 vessels disease; 3VD: 3 vessels disease; EF: ejection fraction; BMI: body mass index; GFR: glomerular filtration rate
